# Epidemiological characteristics and influencing factors of scrub typhus in Jiangxi Province

**DOI:** 10.1186/s13071-025-06908-7

**Published:** 2025-07-05

**Authors:** Yanwu Nie, Shu Yang, Qi Yao, Xiaobo Liu, Baojun Zhang, Yuanan Lu, Yisheng Zhou, Lei Wu, Hui Li

**Affiliations:** 1 School of Public Health, Jiangxi Provincial Key Laboratory of Disease Prevention and Public Health, Jiangxi Medical College, Nanchang University, Nanchang, 330006 China; 2 Jiangxi Provincial Health Commission Key Laboratory of Pathogenic Diagnosis and Genomics of Emerging Infectious Diseases, Nanchang Center for Disease Control and Prevention, Nanchang, 330006 China; 3 National Key Laboratory of Intelligent Tracking and Forecasting for Infectious Diseases, National Institute for Communicable Disease Control and Prevention, Chinese Center for Disease Control and Prevention, Beijing, 102206 China; 4 School of Public Health, Zhejiang Chinese Medical University, Hangzhou, 310053 China; 5 Office of Public Health Studies, University of Hawaii at Manoa, Honolulu, HI 96822 USA

**Keywords:** Scrub typhus, Epidemiological characteristics, Influencing factors, Meteorological factors, Socioeconomic factors, Geographically weighted regression

## Abstract

**Background:**

The large-scale outbreaks of scrub typhus, coupled with the discovery of this vector-borne disease in new regions, indicate that the disease discovery of this vector-borne disease in new regions, indicate that the disease This study aimed to explore the epidemiological characteristics of scrub typhus (ST) in Jiangxi Province and to examine the impacts of meteorological, socioeconomic, and land-cover factors on its incidence.

**Methods:**

Data on reported cases of ST in Jiangxi from 2014 to 2023 were collected. The spatial trend of ST was analyzed via the standard deviation ellipse method. On the basis of the 2022 spatial data, global regression analysis was conducted via ordinary least squares (OLS), whereas local regression analysis was conducted via geographically weighted regression (GWR). The geodetector approach was used to identify the dominant influencing factors and assess the interactions among them.

**Results:**

From 2014 to 2023, the average annual incidence of ST in Jiangxi was 2.025 per 100,000 people, with the peak incidence reported between June and November. Cases were more prevalent among females, with the majority of cases occurring in individuals aged 40–84 years. Farmers represented the most affected occupational group, accounting for 8404 cases (92.06%). The spatial distribution of ST showed an expanding trend across the province. The risk factors identified included elevation, gross domestic product (GDP) per capita, the percentage of agricultural GDP, temperature, and relative humidity. Conversely, a higher percentage of forestry GDP was found to be a protective factor. The effects of these variables exhibited sustained spatial heterogeneity across different regions. The GDP per capita, percentage of forestry GDP, and elevation emerged as the dominant influencing factors. All interactions among variables were enhancement types, primarily characterized by bifactor enhancements.

**Conclusions:**

The incidence of ST in Jiangxi is expanding geographically and is affected by a combination of environmental, socioeconomic, and climatic factors. Strengthening public awareness and preventive measures, particularly in high-incidence areas and among vulnerable populations, is recommended to increase the effectiveness of ST control and prevention efforts.

**Graphical abstract:**

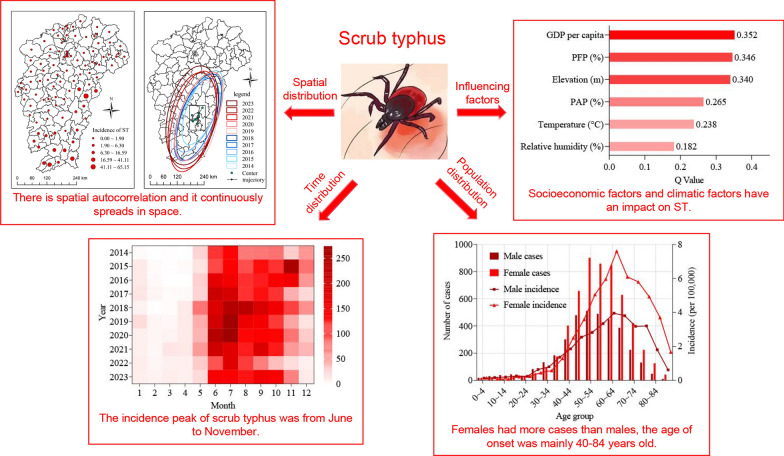

**Supplementary information:**

The online version contains supplementary material available at 10.1186/s13071-025-06908-7.

## Background

Scrub typhus (ST), also known as tsutsugamushi disease, is a zoonotic infectious disease caused by *Orientia tsutsugamushi*, *Candidatus Orientia chuto* [1], and *Candidatus Orientia chiloensis* [2]*.* This disease is transmitted to humans through the bites of chigger mite larvae that parasitize the surfaces of rodent hosts [3]. Characteristic clinical manifestations include high fever, pathognomonic eschar (often accompanied by ulceration), and rash [4]. According to the World Health Organization (WHO), over 55% of the global population currently resides in areas where ST is endemic [5], with an estimated 1 million new cases annually and more than 1 billion people at risk of infection [4]. In recent decades, the incidence and geographical spread of ST have significantly increased, making it a growing public health concern [6]. In addition, a meta-analysis on the global seroprevalence of scrub typhus revealed that the overall seroprevalence rate (95% confidence interval) was 24.93% (23.27–26.60%), highlighting the importance of this neglected disease as a public health issue [7].

The transmission of ST is significantly influenced by the distribution of vector chigger mites and their rodent hosts, both of which are affected by environmental and socioeconomic factors. Ongoing socioeconomic development has significantly transformed occupational structures, altered the balance between natural and built environments, and modified both the scope and patterns of human activities. These anthropogenic changes collectively impact the distribution of chigger mites and their rodent hosts, consequently influencing the dynamics of ST transmission [8, 9]. Previous studies have established an association between the incidence of ST and meteorological as well as socioeconomic variables [10–13]. Li et al. [14] used maximum entropy-based ecological niche modeling and identified the normalized difference vegetation index (NDVI) as the most influential factor, alongside land cover type, population density, temperature, wind speed, and atmospheric pressure. Similarly, Wu et al. [15] reported that certain land cover types and meteorological conditions were key determinants of ST incidence. However, studies on the impact of socioeconomic development on ST remain limited and often yield inconsistent findings. For example, Zheng et al. [16] employed a panel Poisson model to study high-prevalence areas in southern China and reported no significant association between the gross domestic product (GDP) and ST. In contrast, Xin et al. [17] used boosted regression tree modeling and determined that GDP was the most important predictor of ST. These inconsistencies highlight the need for further investigations into the effects of meteorological, socioeconomic, and land cover factors—particularly socioeconomic factors—on ST incidence.

The aim of this study was to analyze the epidemiological characteristics of ST in Jiangxi from 2014 to 2023 and to assess the influence of meteorological, socioeconomic, and land cover factors on its incidence. The findings are intended to serve as a scientific basis for enhancing the prevention and control of ST.

## Methods

### Study location

Jiangxi Province is located in the southeastern part of mainland China, spanning latitudes 24° 29′–30° 04′ N and longitudes 113° 34′–118° 28′ E. It covers a total area of 166,900 square kilometers, accounting for approximately 1.74% of China’s land area. Situated in the subtropical zone, Jiangxi experiences a distinct monsoon climate with four clearly defined seasons. At the end of 2023, Jiangxi governed 100 county-level administrative divisions and had a resident population of approximately 45.15 million. The geographic scope of our study is presented in Supplementary Fig. S1.

### Data summary

The 2014–2023 data on ST in Jiangxi were obtained from the Chinese Center for Disease Control and Prevention (CDC). The original data contained information on sex, age, occupation, date of ST onset, and address code. The incidence data of 100 districts and counties in Jiangxi in 2022 were summarized on the basis of address codes. The total population of each age group was obtained from the Jiangxi Provincial Statistical Yearbook to calculate the incidence rate of each age group for males and females. The sources for meteorological data, socioeconomic data, and land cover data and the variables included are presented in Supplementary Table S1.

### OLS

The OLS model is a commonly used traditional linear regression model that only averages or globally estimates the parameters but fails to capture the spatial nonstationarity of each parameter [18]. The model is calculated as follows:

$$ Y_{i} = \beta_{0} + \sum\nolimits_{k} {\beta_{k} X_{ik} + \varepsilon_{i} } $$

where $Y_{i}$ denotes the dependent variable at the *i*th sample point, $\beta_{0}$ denotes the intercept of the linear regression equation, $\beta_{k}$ denotes the regression coefficient of the *k*th independent variable, $X_{ik}$ denotes the *k*th independent variable at the *i*th sample point, and $\varepsilon_{i}$ denotes the random error.

### Geographical weighted regression

Geographically weighted regression (GWR) is a spatially varying coefficient regression model used to reveal the differences in regional effects across each spatial area [19]. It assumes that the regression coefficients are functions of the geographical location of a region. Each spatial unit estimates local parameters by using the data of its neighboring units through weighted least squares to obtain the regression coefficients, which enables the differences in the spatial structure of the results to be better displayed [20]. The formula for the model is as follows:

$$ Y_{i} = \beta_{0} \left( {u_{i} ,\upsilon_{i} } \right) + \sum\nolimits_{k} {\beta_{k} \left( {u_{i} ,\upsilon_{i} } \right)} X_{ik} + \varepsilon_{i} $$

where $\left( {u_{i} ,\upsilon_{i} } \right)$ is the geographic location coordinate of the *i*th spatial unit, $\beta_{k} \left( {u_{i} ,\upsilon_{i} } \right)$ is the regression coefficient that varies with spatial geographic location, and $\varepsilon_{i}$ is the random error term. In this study, the parameters were standardized, the adaptive bisquare function was selected as the kernel function, and the cross-validation (CV) method was selected for bandwidth optimization. Akaike’s information criterion corrected (AICc) and adjusted R^2^ values were calculated to evaluate the model fit.

### Geodetector

Geodetectors, which include factor detectors, risk detectors, ecological detectors, and interaction detectors, are based on the spatial stratified heterogeneity theory to determine the degrees of correlation between independent variables and dependent variables at the same spatial scale [21]. Factor and interaction detectors were used in this study.

- 1) *Factor detector* A factor detector is used to detect the strength of the effect of each factor on the spatial ST distribution in Jiangxi. In this study, we use the statistic *q* value, which is between 0 and 1. The larger the *q* value is, the stronger the driving factor in explaining the spatial distribution of ST in Jiangxi.
- 2) *Interaction detector* An interaction detector was used to analyze the interaction between the driving factors on ST and to identify the strength, whether it was linear or not, and the direction. The interaction types of the two driving factors are presented in Table 1.

**Table 1:** Interactions between variables

Expression	Interaction
*q*(*x*, *y*) < Min(*q*(*x*), *q*(*y*))	Nonlinear weaken
Min(*q*(*x*), *q*(*y*)) < *q*(*x*, *y*) < Max(*q*(*x*), *q*(*y*))	Unitary-nonlinear weaken
*q*(*x*, *y*) > Max(*q*(*x*), *q*(*y*))	Bifactor enhancement
*q*(*x*, *y*) = *q*(*x*) + *q*(*y*)	Independent
*q*(*x*, *y*) > *q*(*x*) + *q*(*y*)	Nonlinear enhancement

### Statistical analysis

In this study, descriptive methods were employed to analyze the epidemic characteristics of ST in Jiangxi, and the spatial distribution trend of ST was analyzed via standard deviation ellipses. Global regression of the influencing factors was performed with a stepwise OLS model, sequentially eliminating all variables with nonsignificant *P* values. Subsequently, local regression was conducted on the significant variables via the GWR model. The geodetector was employed to assess the explanatory powers of individual factors and their interactions with the spatial distribution of ST. Visualization was performed via ArcGIS (10.7) and GraphPad (version 9.0), whereas statistical analyses were conducted via GWR (4.0) and R (4.4.1).

## Results

### Time distribution characteristics

**Fig. 1:**
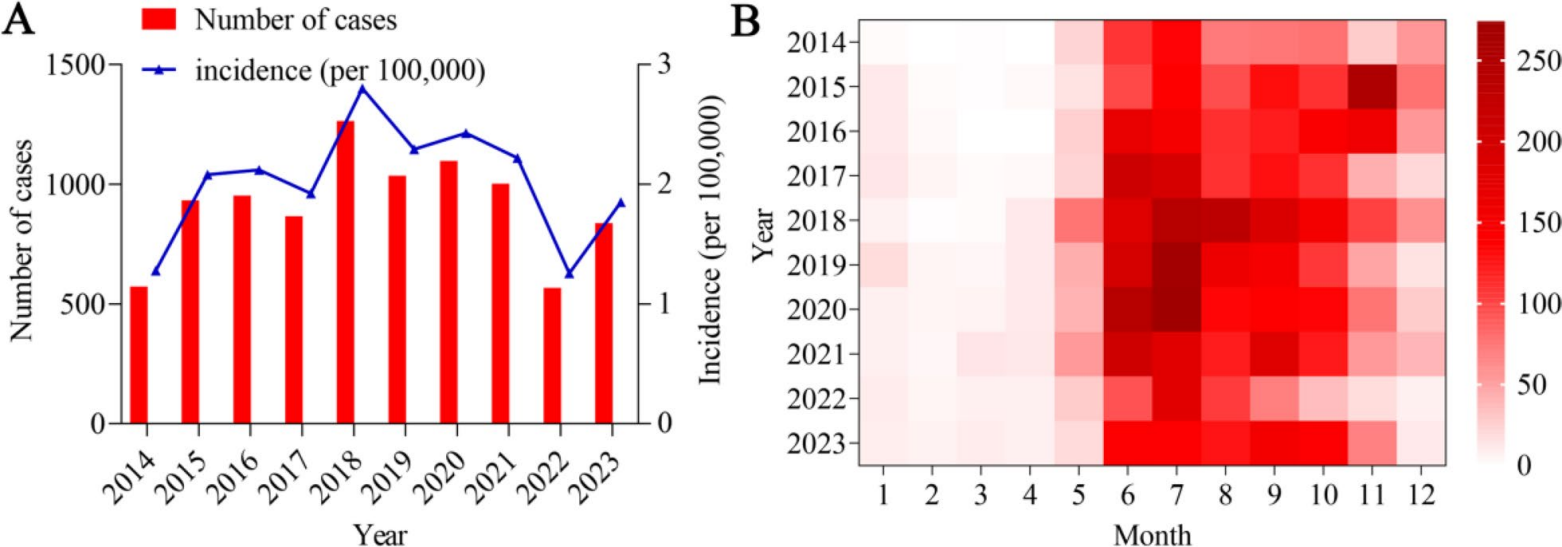
Characteristics of the temporal distribution of ST in Jiangxi, 2014–2023. (A) Year Distribution plot. (B) Heatmap

A total of 9129 ST cases were reported in Jiangxi from 2014 to 2023. Figure 1A shows the yearly distribution of ST in Jiangxi from 2014 to 2023, with the largest number of cases reported in 2018 (1264 cases), the lowest number of cases reported in 2022 (567 cases), and an average annual incidence rate of 2.025/100,000 cases per year. Figure 1B shows a heatmap of the ST incidences in Jiangxi in different years and months from 2014 to 2023. June to November are the months with the highest ST incidences in Jiangxi.

### Population distribution characteristics

**Fig. 2:**
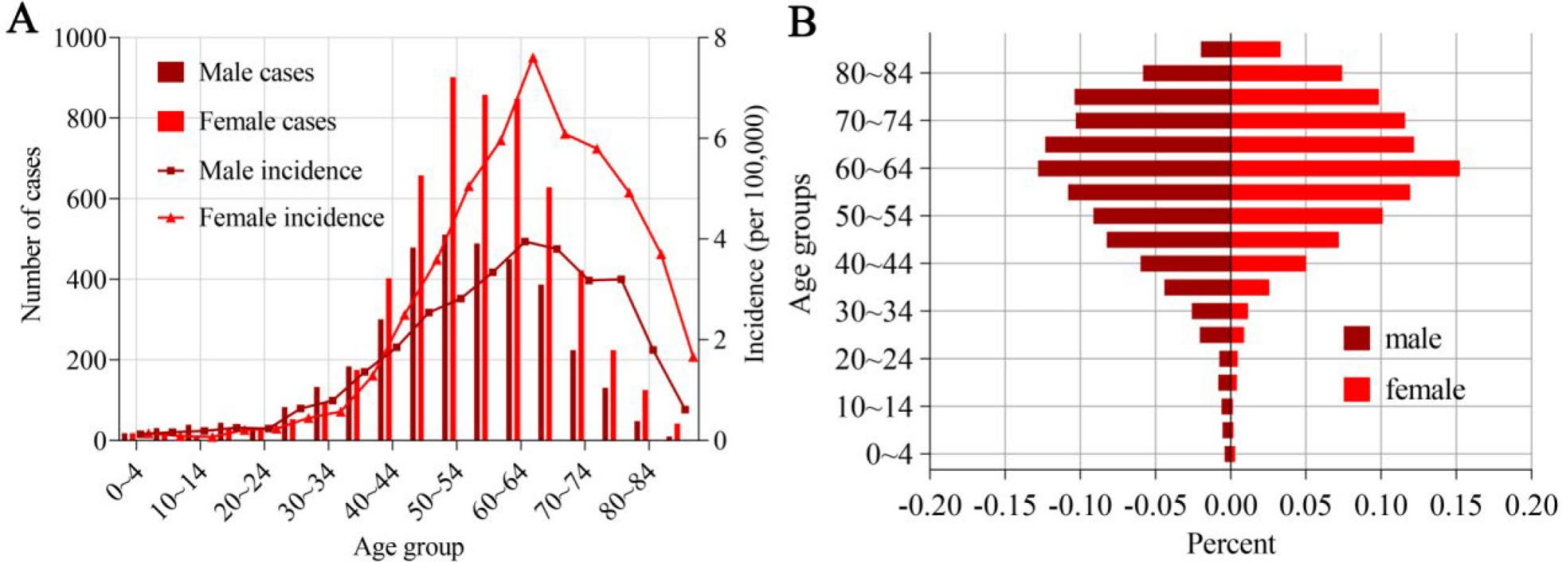
Characteristics of the ST age distribution in Jiangxi, 2014–2023. (A) Dual y-axis plot. (B) Proportional composition plot

In Jiangxi, ST is prevalent among farmers. The number of patients who were farmers was 8404 (92.06%). From 2014 to 2023, 3594 male patients (39.37%) and 5535 female patients (60.63%) with ST were reported in Jiangxi. The gender ratio of males to females is 0.649. Figure 2 shows the characteristics of the age distribution of patients with ST. Patients were from all age groups. The youngest scrub typhus patient was 0 years old, and the oldest was 94 years old. ST is mainly concentrated in the age range of 40–84 years. Among these populations, both male and female populations have the highest incidence rates at ages of 60–64 years.

### Spatial distribution characteristics

**Fig. 3:**
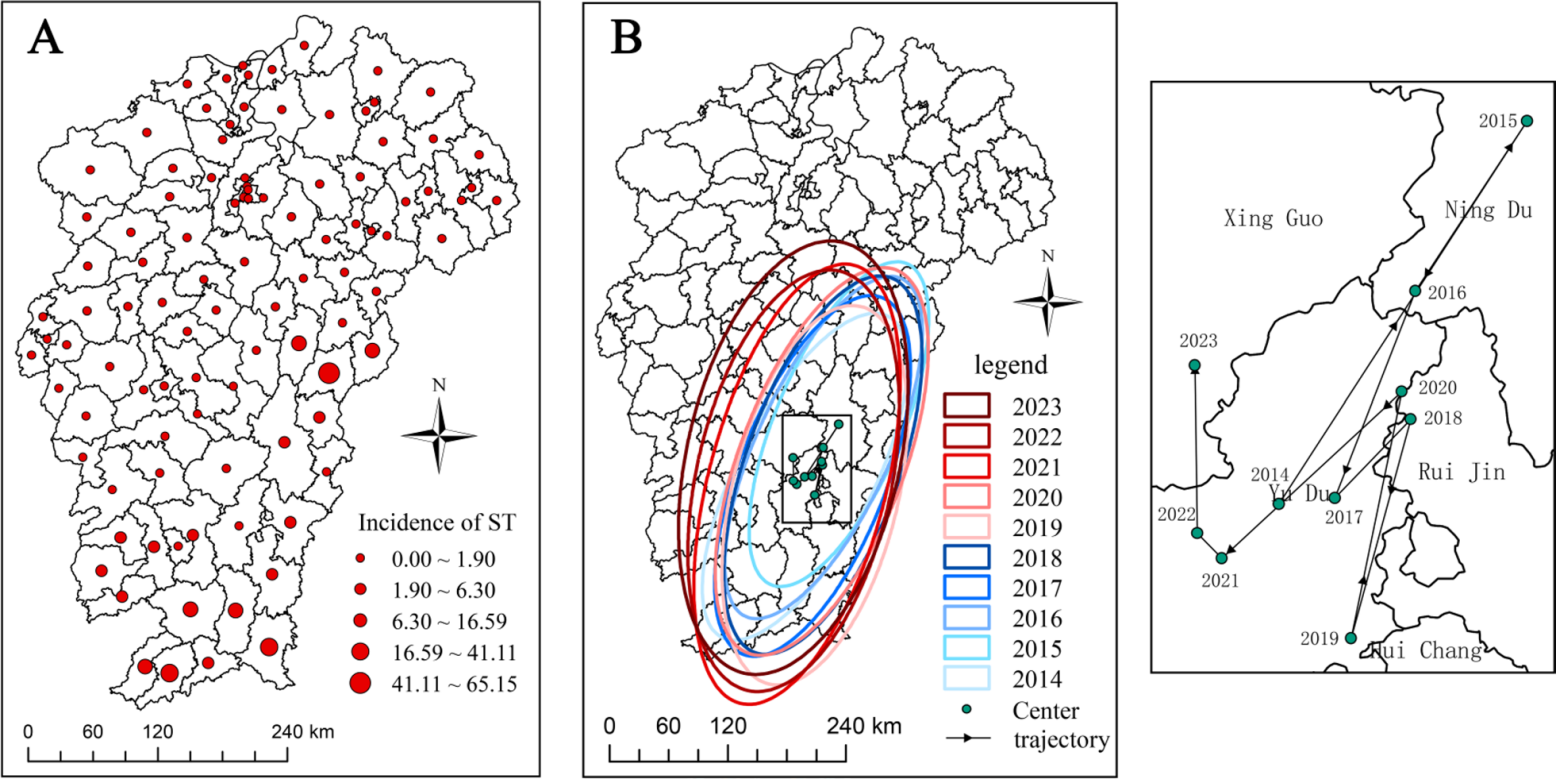
Characteristics of the spatial distribution of ST in Jiangxi, 2014–2023. (A) Spatial distribution map. (B) Standard deviation ellipses and center migration trajectory map

Figure 3A shows the spatial distribution of ST in Jiangxi from 2014 to 2023. We found that Ganzhou and Fuzhou are the cities with high incidences of ST. Figure 3B shows the standard deviation ellipses and center migration trajectory maps of ST from 2014 to 2023, and Supplementary Table S2 shows the parameter list for the standard deviation ellipses. We found that the areas of the standard deviation ellipses for ST in Jiangxi showed an expanding trend, expanding from 39,580,000 km^2^ in 2014 to 76,150,000 km^2^ in 2023. In addition, the changes in the long and short axes of the standard deviation ellipses indicated that the ST incidences in Jiangxi during the period of 2014–2023 showed an expanding trend in the east–west and north–south directions. The center migration trajectory indicates that the center of the ST incidences in Jiangxi has been in Ganzhou City.

### Regression analysis

There was global autocorrelation (Molan I = 0.200, *P* = 0.004) in the incidence of ST in Jiangxi in 2022, indicating spatial aggregation of ST. The multicollinearity results revealed that there was no high degree of multicollinearity among the variables. Table 2 shows the results of OLS modeling for ST in Jiangxi in 2022, from which it can be seen that elevation, GDP per capita, percentage of agricultural GDP, temperature, and relative humidity are risk factors for ST. The percentage of forestry GDP is a protective factor for ST.

**Table 2:** Results of OLS modeling of factors affecting ST in Jiangxi Province in 2022

Variables	Coefficient	Standard error	*t*	*P*	VIF
Elevation (m)	0.443	0.141	3.155	0.002	3.230
GDP per capita	0.416	0.111	3.747	< 0.001	2.016
Percentage of agricultural GDP	0.500	0.101	4.960	< 0.001	1.665
Percentage of gross forestry GDP	− 0.278	0.110	−2.523	0.012	1.990
Temperature (°C)	0.193	0.095	2.028	0.043	1.484
Relative humidity (%)	0.285	0.131	2.180	0.029	2.799

GDP: Gross *Domestic Product,* VIF: *Variance inflation factor*

**Fig. 4:**
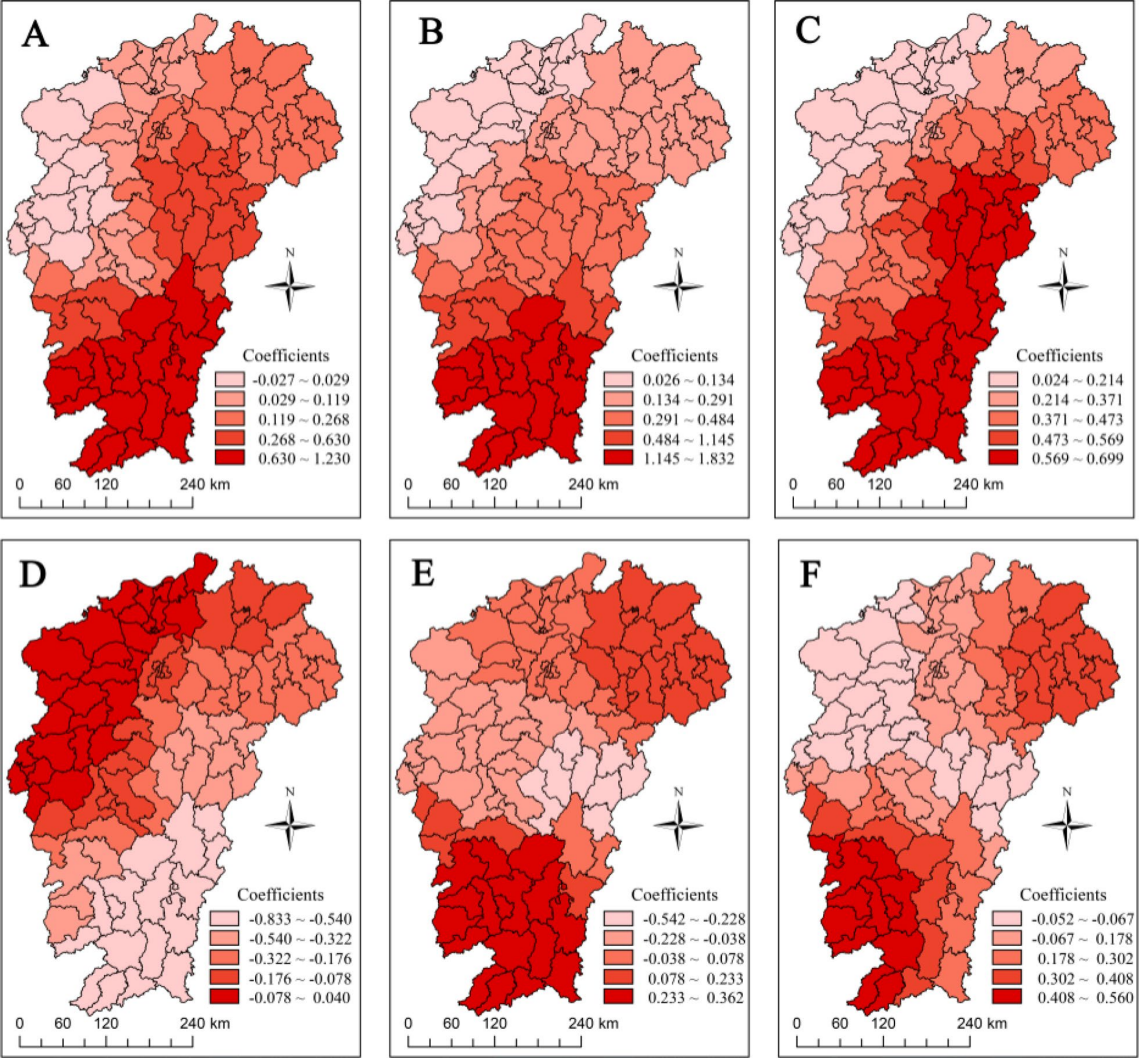
Distribution of local regression coefficients of the GWR model for factors affecting ST in Jiangxi in 2022. **A** Elevation. **B** GDP per capita. **C** Percentage of agricultural GDP. **D** Percentage of forestry GDP. **E** Temperature. **F** Relative humidity

Table 3 presents the distribution of the regression coefficients of the GWR model, and we find that the mean values of the regression coefficients of the five factors are consistent with the results obtained via OLS. Further visualization of the regression coefficients of the GWR results via ArcGIS revealed spatial heterogeneity in the effects of the respective variables on ST in different spatial units (Fig. 4).

**Table 3:** Distribution of regression coefficients of GWR model for factors affecting ST in Jiangxi in 2022

Variables	Mean	Standard deviation	Minimum	Median	Maximum
Elevation (m)	0.322	0.344	−0.027	0.201	1.230
GDP per capita	0.487	0.538	0.026	0.246	1.832
Percentage of agricultural GDP	0.407	0.200	0.024	0.425	0.699
Percentage of gross forestry GDP	−0.233	0.241	−0.833	−0.154	0.040
Temperature (°C)	0.051	0.187	−0.542	0.032	0.362
Relative humidity (%)	0.209	0.161	−0.052	0.158	0.560

A comparison of the main diagnostic indicators of the two models revealed that the AICc and residual sum of squares (RSS) values of the GWR model were smaller than the corresponding indicators of the OLS model, and the adjusted *R*^2^ value was larger than that of the OLS model, suggesting that the fit of the GWR model was significantly better than that of the OLS model (Table 4). The adjusted *R*^2^ value that was estimated by the GWR model in this study was 0.630, which means that the model can explain 63% of the total variation in the incidence of ST in Jiangxi.

**Table 4:** Comparison of OLS and GWR models

Model	*R*^2^	Adj. *R*^2^	AICc	RSS
OLS	0.431	0.395	244.917	56.863
GWR	0.700	0.630	213.138	29.962

OLS: *Ordinary Least Squares*, GWR: *Geographically Weighted Regression*, AICc: *Akaike Information Criterion corrected*, RSS: *Residual Sum of Squares*

### Geodetector

**Fig. 5:**
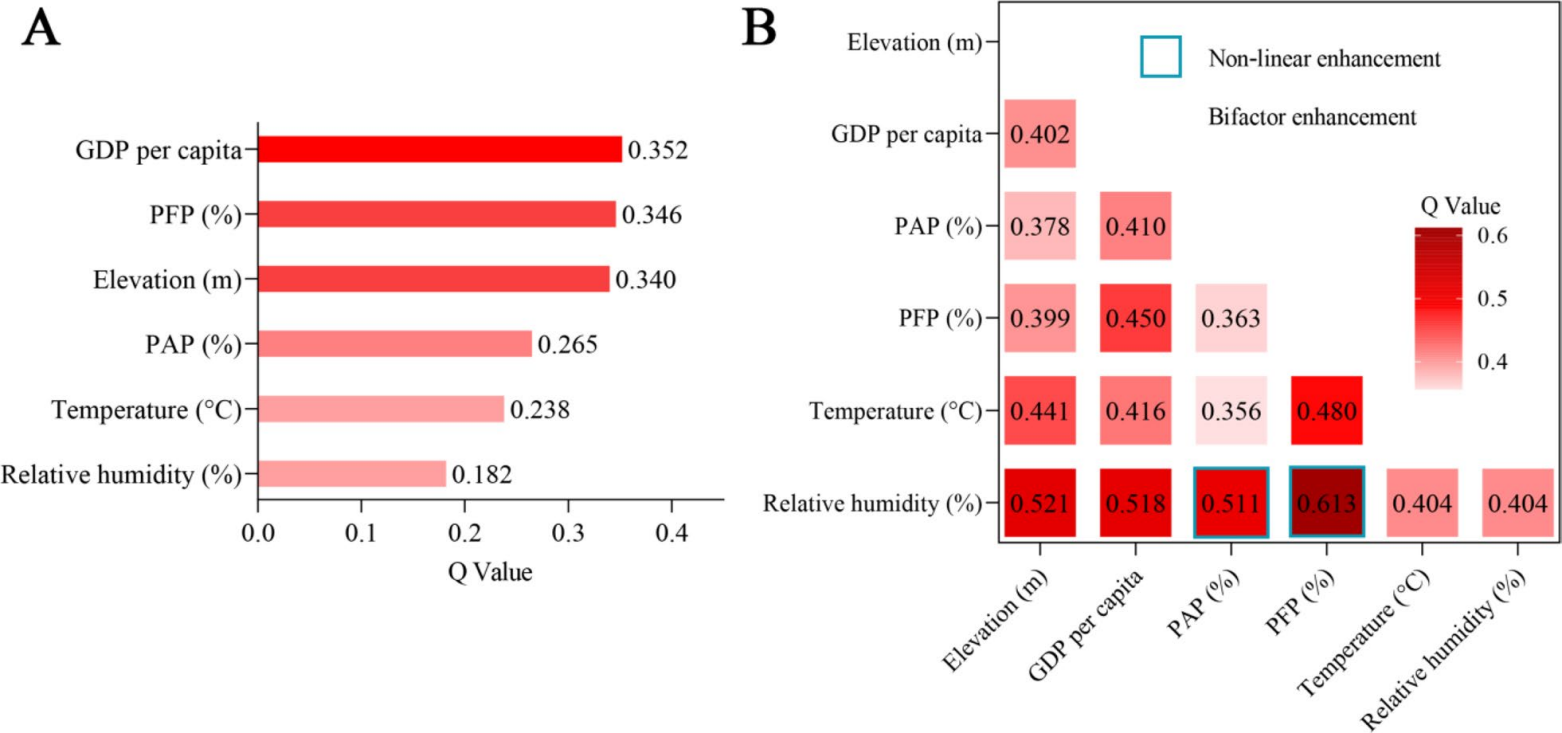
Geodetector results for 2022. **A** Factor detection. **B** Interaction detection. *PFP* Percentage of forestry GDP. *PAP* Percentage of agricultural GDP

Factor detection was used to calculate the strength of the influence of various factors on ST (Fig. 5A). All six factors passed the significance test at the 0.05 level. The explanatory powers of the spatial variance in ST in descending order were per capita GDP (0.352) > percentage of forestry GDP (0.346) > elevation (0.340) > percentage of agricultural GDP (0.265) > temperature (0.223) > relative humidity (0.182), with per capita GDP, percentage of forestry GDP, and elevation explaining a relatively large amount of the spatial variance in ST. Interaction detection was used to calculate the interactions of the influencing factors on ST (Fig. 5B). We found that the types of interactions between factors all increased, with two-factor increases dominating, and that ST in the Jiangxi region was caused by the combined effects of multiple factors.

## Discussion

Scrub typhus is a naturally occurring acute febrile illness that is influenced by climate conditions, socioeconomic development, and land cover characteristics. Despite growing interest, research on the relationship between socioeconomic factors and ST remains limited, and the findings have often been inconsistent owing to methodological constraints and varying analytical depths. This study aims to analyze the epidemiological characteristics of ST in Jiangxi and to comprehensively assess the impacts of meteorological, socioeconomic, and land cover factors on its incidence.

The average annual incidence of ST in Jiangxi is 2.025 per 100,000, with peak cases occurring from June to November and almost no cases occurring from January to April each year. These findings indicate that ST in Jiangxi exhibits distinct seasonal patterns [22]. Previous research has categorized the outbreaks that peak from June to August as the summer type, whereas those occurring from September to November are classified as the autumn type [23]. The outbreak peak in Jiangxi occurred between June and November, representing a summer–autumn pattern. This phenomenon may be attributed to the presence of diverse vector chigger mite species. The ST epidemic durations vary depending on the vectors of the chigger mite species involved. For example, *Leptotrombidium deliense* and *Leptotrombidium scutellare* cause summer-type and autumn-type epidemics, respectively [24]. Furthermore, the abundance of food resources during the harvest season supports rodent host survival, whereas increased human outdoor activity during summer and fall contributes to increased ST incidence during these periods [25].

In this study, we observed a notable expansion trend in the endemic ST area in Jiangxi, extending both east–west and north–south. This finding is consistent with those of previous studies [15, 26]. Environmentally harmful activities, such as excessive greenhouse gas emissions and changes in land use, have intensified global warming, leading to shifts in the distributions of host habitats and local flora [27–29]. In addition, human movements significantly contribute to the spread of ST. With rapid economic development and changes in the transportation infrastructure, the cross-regional transmission of infectious diseases has become increasingly concerning [30]. Therefore, it is essential to implement appropriate measures during epidemic seasons, including reducing rodent and chigger densities in affected areas or enhancing personal protection and health education for travelers entering these regions. Simultaneously, prolonged exposure to farmland and grassland should be minimized to reduce the risk of chigger bites.

The population distribution analysis revealed that the primary incidence of ST in Jiangxi occurred among female farmers aged 40–84 years, a pattern consistent with the demographic trends reported in other regions of China [31, 32]. This trend may be explained by the socioeconomic dynamics in suburban and rural areas, where young males often migrate for employment opportunities, leaving females and elderly individuals to manage agricultural work. As a result, these groups are more frequently present in environments such as meadows, fields, and wooded areas, increasing their risk of encountering ST vectors and contributing to the higher incidence observed among females and older adults in rural communities.

A study conducted in Taiwan, China [33] revealed that the risk of ST increases with elevation, a finding that aligns with our observations. However, the specific mechanisms by which elevation influences ST transmission remain unclear. Some studies have proposed that elevation may act as a proxy for a combination of environmental factors that collectively affect disease risk [33]. However, another study led by Acharya et al. presented contrasting conclusions [34], suggesting a potentially nonlinear relationship between elevation and ST incidence. For example, at lower elevations, increased vegetation density and higher relative humidity may favor the survival and reproduction of mites and their hosts [33]. Conversely, as the elevation increases beyond a certain point, both temperatures and vegetation cover decline, leading to less favorable conditions for mite and rodent populations [14]. These complexities underscore the need for further research to clarify the relationship between elevation and chigger dynamics.

Jiangxi Province exhibits significant regional disparities in both economic development and health care resource distributions, demonstrating a distinct north-high south-low spatial pattern. The influence of economic factors on ST remains debated. While several previous studies have reported no association between GDP and ST incidence [13, 14, 16], the present study revealed a positive correlation between per capita GDP and ST. Further analysis of the economic sector impacts revealed that a higher proportion of agricultural GDP is linked to increased ST risk, whereas a greater share of forestry products is associated with reduced risk. Numerous studies have shown that ST primarily affects farmers [35, 36]. A large agricultural share suggests a predominantly agrarian economy, with more individuals working and moving through grasslands, fields, and wilderness, environments where contact with ST mites is more likely, contributing to higher incidence rates. A lower per capita GDP value typically indicates limited government investment in public health and education [37], resulting in poorer health literacy and insufficient awareness of ST transmission risks. Moreover, economically disadvantaged areas often lack effective preventive measures, leading to a higher incidence of scrub typhus.

This study revealed positive associations between temperature, relative humidity, and the incidence of ST, which is consistent with the findings of previous studies [38, 39]. These associations may be attributed to the fact that temperature and humidity are critical for mite development and reproduction. Warm and humid conditions support mite growth, whereas lower temperatures can extend their developmental cycle and increase mortality before reproduction [40]. Relative humidity also plays a key role in determining mite density, as mites depend on water vapor for their survival [41]. The presence of ST has also been identified in the relatively humid Tierra del Fuego ecoregion [42]. In addition, some studies suggest a nonlinear relationship between temperature, humidity, and chigger activity. Thus, further investigations using time series analyses are needed to better understand the impacts of meteorological factors on chiggers.

This study has several limitations. First, the data were obtained from a passive infectious disease surveillance system, which may be susceptible to underreporting and misclassification. To minimize misdiagnosis and underreporting, it is essential to improve the accuracy of ST diagnoses, optimize the electronic reporting process, and strengthen public education and awareness campaigns. Second, the study did not account for the spatial distribution of vector chiggers and their host animals, which may influence ST transmission. Future research will incorporate field-based rodent and vector surveillance to improve the understanding of their roles in the epidemiology of ST. Nevertheless, this type of modeling approach may represent a more cost-effective alternative to vector or rodent surveillance and can support predictive efforts, public education, and awareness in endemic areas.

## Conclusions

The coverage of scrub typhus in Jiangxi Province has shown a trend of continuous expansion. Scrub typhus is influenced by a combination of factors, including meteorological and socioeconomic factors. Notably, the per capita GDP, the percentage of forestry output value, and altitude are the dominant factors affecting the incidence of scrub typhus. We recommend enhancing training in the diagnosis and treatment of infectious diseases in areas with high incidence rates and among high-risk populations and increasing public awareness and knowledge about disease prevention.

## Supplementary information

Supplementary material 1.

## Data Availability

The data mentioned in our study came from national databases, which were unsuitable for publicly sharing without their permission. Interested parties can apply for the ST data by contacting the Data-center of China Public Health Science (https://www.phsciencedata.cn/Share/edtShareNew.jsp?id=39304), and apply for the meteorological data from the China Meteorological Data Sharing Service System (https://data.cma.cn/dataService/cdcindex/datacode/A.0012.0001/show_value/normal.html).
