# Supplementary material for: Epidemiological characteristics and influencing factors of scrub typhus in Jiangxi Province
Source: Parasit Vectors. 2025 Jul 5;18:260. doi: 10.1186/s13071-025-06908-7 (PMC12228367; doi:10.1186/s13071-025-06908-7)
Supplement: Supplementary file 1 — Supplementary material 1. [file 13071_2025_6908_MOESM1_ESM.docx]

**
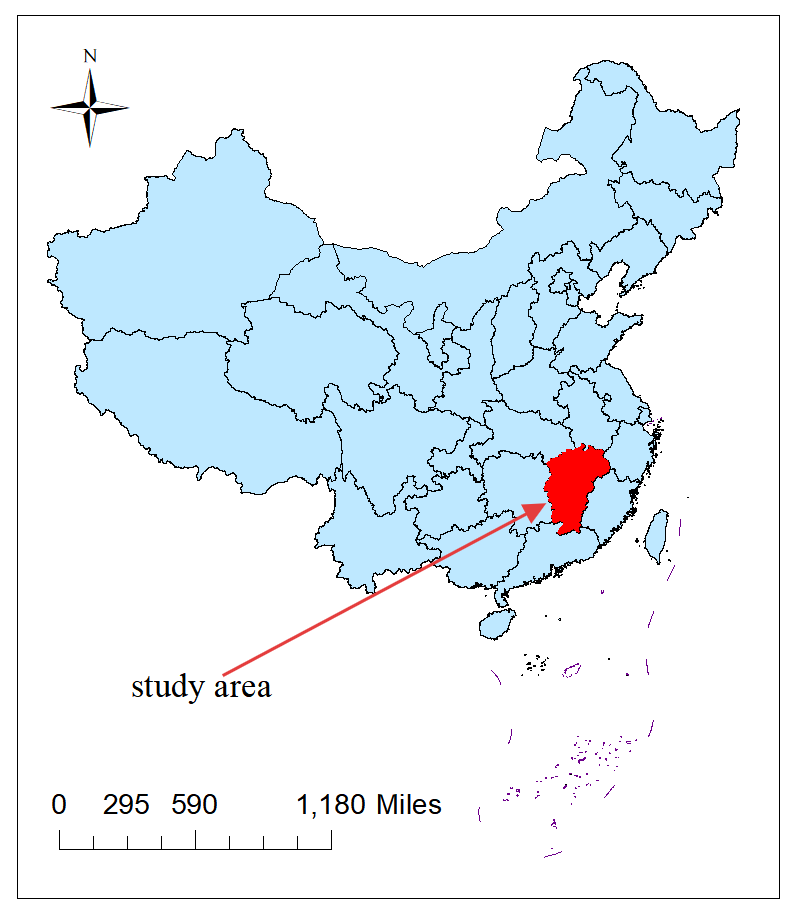
**

**Figure S1** The research area of this study

**Table S1** Sources of data on factors influencing ST

| Variable category | Variable | Sources |
| --- | --- | --- |
| Socioeconomic | Urbanization rate(%) | Statistical yearbooks for various cities in |
|  | Population density |  |
|  | GDP per capita |  |
|  | percentage of agricultural GDP (%) |  |
|  | percentage of forestry GDP (%) |  |
| Climate | Temperature (°C) | Resource and Environment Science  and Data Center  ([www.resdc.cn](http://www.resdc.cn/)) |
|  | Relative humidity (%) |  |
|  | Wind speed (m/s) |  |
|  | Precipitation (mm) |  |
|  | Pressure (kpa) |  |
| land cover | Farmland area proportion (%) | Zenodo  (https://zenodo.org/records/12779975) |
|  | Forest area proportion (%) |  |
|  | Water area proportion (%) |  |
|  | Elevation (m) | Geospatial data cloud ([www.gscloud.cn](http://www.gscloud.cn/)) |
|  | NDVI | Resource and Environment Science and Data Center ([www.resdc.cn](http://www.resdc.cn/)) |

**Table S2** Standard deviation ellipse parameters of ST in Jiangxi, 2014-2023

| Year | Ellipse area (10,000 km^2^) | Long axis (km) | Short axis (km) | Angle (degree) |
| --- | --- | --- | --- | --- |
| 2014 | 3.958 | 183.485 | 68.672 | 27.430 |
| 2015 | 3.590 | 176.873 | 64.609 | 23.042 |
| 2016 | 4.008 | 219.151 | 67.590 | 24.866 |
| 2017 | 4.585 | 194.347 | 75.108 | 21.368 |
| 2018 | 4.970 | 202.308 | 78.202 | 19.910 |
| 2019 | 5.217 | 197.167 | 84.228 | 13.762 |
| 2020 | 5.466 | 208.501 | 83.452 | 21.145 |
| 2021 | 6.505 | 229.768 | 90.129 | 14.657 |
| 2022 | 6.783 | 217.818 | 99.136 | 12.338 |
| 2023 | 7.615 | 224.298 | 108.079 | 12.861 |
